# Conditional Deletion of Dicer in Adult Mice Impairs Skeletal Muscle Regeneration

**DOI:** 10.3390/ijms20225686

**Published:** 2019-11-13

**Authors:** Satoshi Oikawa, Minjung Lee, Takayuki Akimoto

**Affiliations:** Faculty of Sport Sciences, Waseda University, Saitama 359-1192, Japan; s-oikawa@aoni.waseda.jp (S.O.); namoonyousun@gmail.com (M.L.)

**Keywords:** Dicer, microRNA, skeletal muscle, muscle regeneration

## Abstract

Skeletal muscle has a remarkable regenerative capacity, which is orchestrated by multiple processes, including the proliferation, fusion, and differentiation of the resident stem cells in muscle. MicroRNAs (miRNAs) are small noncoding RNAs that mediate the translational repression or degradation of mRNA to regulate diverse biological functions. Previous studies have suggested that several miRNAs play important roles in myoblast proliferation and differentiation in vitro. However, their potential roles in skeletal muscle regeneration in vivo have not been fully established. In this study, we generated a mouse in which the *Dicer* gene, which encodes an enzyme essential in miRNA processing, was knocked out in a tamoxifen-inducible way (iDicer KO mouse) and determined its regenerative potential after cardiotoxin-induced acute muscle injury. *Dicer* mRNA expression was significantly reduced in the tibialis anterior muscle of the iDicer KO mice, whereas the expression of muscle-enriched miRNAs was only slightly reduced in the Dicer-deficient muscles. After cardiotoxin injection, the iDicer KO mice showed impaired muscle regeneration. We also demonstrated that the number of PAX7^+^ cells, cell proliferation, and the myogenic differentiation capacity of the primary myoblasts did not differ between the wild-type and the iDicer KO mice. Taken together, these data demonstrate that Dicer is a critical factor for muscle regeneration in vivo.

## 1. Introduction

Adult skeletal muscle has a remarkable regenerative capacity. After muscle injury, the resident muscle stem cells leave their quiescent state and begin to proliferate. These cells ultimately differentiate into multinucleated myotubes, which fuse with existing damaged myofibers [1]. Muscle regeneration is impaired by treatment with a mitosis inhibitor, colchicine, which inhibits cellular proliferation during regeneration [2]. Primary satellite cells (SCs) derived from mutant mice lacking PAX7, a marker of SCs, showed reduced proliferation and fewer myosin heavy chain (MyHC)-positive myotubes [3,4]. The adult *Pax7*-mutant mice also showed impaired injury-induced muscle regeneration [3]. These findings indicate that the proliferation and myogenic differentiation of resident muscle stem cells are necessary for muscle regeneration.

MicroRNAs (miRNAs) are small noncoding RNAs that repress the expression of their target genes at the posttranscriptional level to control diverse biological functions [5]. These small RNAs can bind to a complementary site, called “seed sequences”, in the 3′ untranslated region (UTR) of a target mRNA, resulting in the degradation or translational repression of that mRNA [5]. miRNAs are transcribed as long primary transcripts, called “primary miRNAs”, by an RNA polymerase II. The transcripts are cleaved by a nuclear ribonuclease III, Drosha, and then exported to the cytoplasm and further cleaved by a cytoplasmic ribonuclease III, Dicer, into double-stranded RNA. This miRNA duplex is loaded onto an Argonaute protein to form a ribonucleoprotein complex called the “RNA-induced silencing complex” [6].

Growing evidence indicates that miRNA-mediated gene silencing plays an important role in skeletal muscle cell growth and myogenic differentiation [7,8,9,10,11]. Mutant mice in which Dicer is inactivated during embryonic myogenesis show a variety of abnormal muscle phenotypes, suggesting that Dicer-mediated miRNA processing plays an essential role in muscle development [12]. An expression profiling analysis showed that three miRNAs, miR-1, miR-133, and miR-206, are abundantly expressed in skeletal muscles [13]. These three miRNAs are transcriptionally regulated by myogenic regulatory factors, which are master transcriptional factors for skeletal muscle cell-fate determination and development, and are upregulated during muscle differentiation [14,15,16,17]. It has been shown that miR-1 and miR-206, which have identical seed sequences, promote myogenesis in vitro, whereas miR-133 promotes myoblast proliferation [7,8]. A previous study also demonstrated that miR-1 and miR-206 regulate apoptosis by repressing *Pax3* gene expression in *Myod*-KO myoblasts [18]. Although these data indicate that miRNAs are important regulators of the proliferation and differentiation of muscle cells in vitro, their potential roles in muscle regeneration in vivo have not been fully established.

In this study, we generated a tamoxifen-inducible conditional *Dicer*-KO (iDicer KO) mouse to deplete all mature miRNAs and analyzed its regenerative capacity during cardiotoxin-induced muscle regeneration. Our data suggest that Dicer-mediated miRNA processing is necessary for skeletal muscle regeneration in vivo.

## 2. Results

### 2.1. Expression of Dicer and miRNAs in iDicer KO Mice

**Figure 1:**
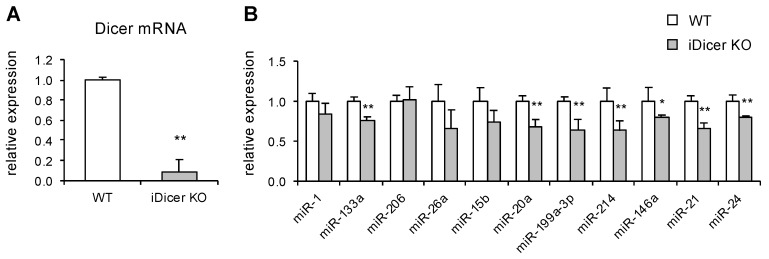
Expression levels of *Dicer* mRNA and muscle-enriched miRNAs in tibialis anterior (TA) muscles of tamoxifen-induced *Dicer* knock-out (iDicer KO) mice. (**A**) Tamoxifen induced a large reduction in *Dicer* mRNA expression in the iDicer KO mice (*n* = 5). (**B**) Expression levels of muscle-enriched miR-1, miR-133a and miR-26a and other miRNAs (miR-15b, miR-20a, miR-199a-3p, miR-214, miR-146a, miR-21 and miR-24) were slightly reduced in TA muscle of the iDicer KO mice by tamoxifen injection (*n* = 3–5). Data are means ± SE, * *p* < 0.05, ** *p* < 0.01.

To investigate the role of miRNAs in muscle regeneration, we first generated a mutant mouse with the tamoxifen-inducible disruption of the *Dicer* gene (iDicer KO). Consistent with our previous data [19], a real-time PCR analysis confirmed the significant reduction in *Dicer* mRNA expression in the tibialis anterior (TA) muscles of the iDicer KO mice (Figure 1A), whereas the expression levels of the muscle-enriched miRNAs miR-1, miR-133a and miR-26a, and other miRNAs (miR-15b, miR-20a, miR-199a-3p, miR-214, miR-146a, miR-21 and miR-24) were modestly reduced in the iDicer KO mice (Figure 1B).

### 2.2. Skeletal Muscle Regeneration Is Impaired in iDicer KO Mice

**Figure 2:**
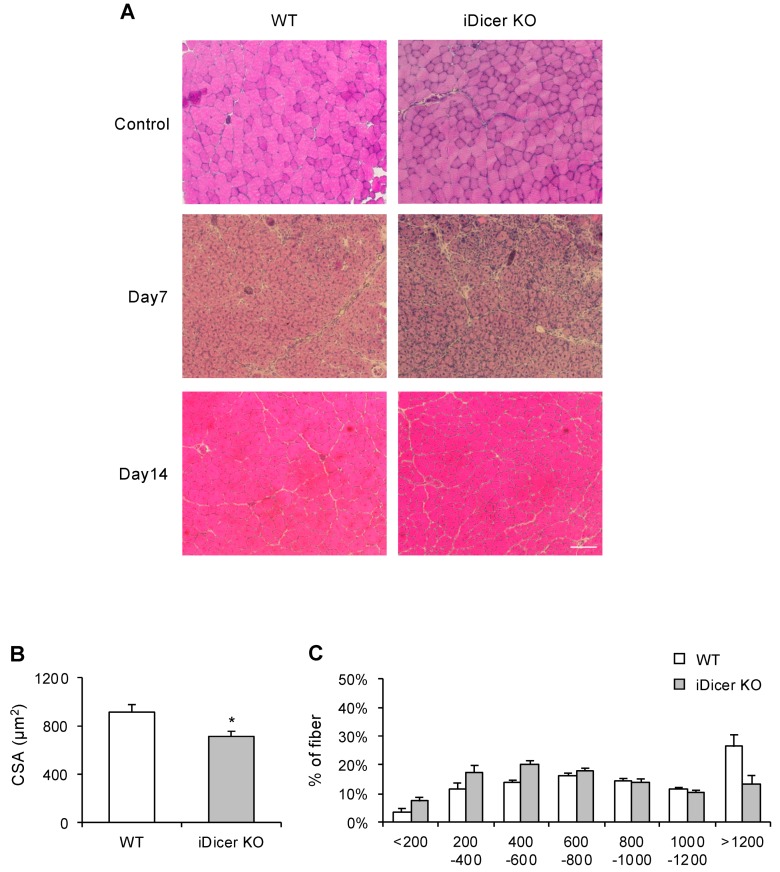
Skeletal muscle regeneration in the iDicer KO mice. (**A**) Representative image of sections of TA muscle stained with hematoxylin–eosin (H&E). Scale bar = 100 µm. (**B**,**C**) Mean cross-sectional area (CSA) of regenerating muscle fibers with central nuclei in the iDicer KO mice was significantly smaller than that in the WT mice (*n* = 7). Data are means ± SE, * *p* < 0.05.

We next determined the regenerative potential of the iDicer KO mice during skeletal muscle regeneration. Wild-type (WT) and iDicer KO mice were injected intramuscularly with cardiotoxin (CTX) to induce muscle injury, and the cross-sectional area (CSA) of the regenerating myofibers was analyzed with hematoxylin–eosin (H&E) staining. Fourteen days after CTX injection, the mean CSA of the regenerating myofibers with central nuclei in the iDicer KO mice was smaller than that in the WT mice (Figure 2A–C).

### 2.3. Inducible Knockout of Dicer Does Not Affect Cell Proliferation or Differentiation of Primary Myoblasts

**Figure 3:**
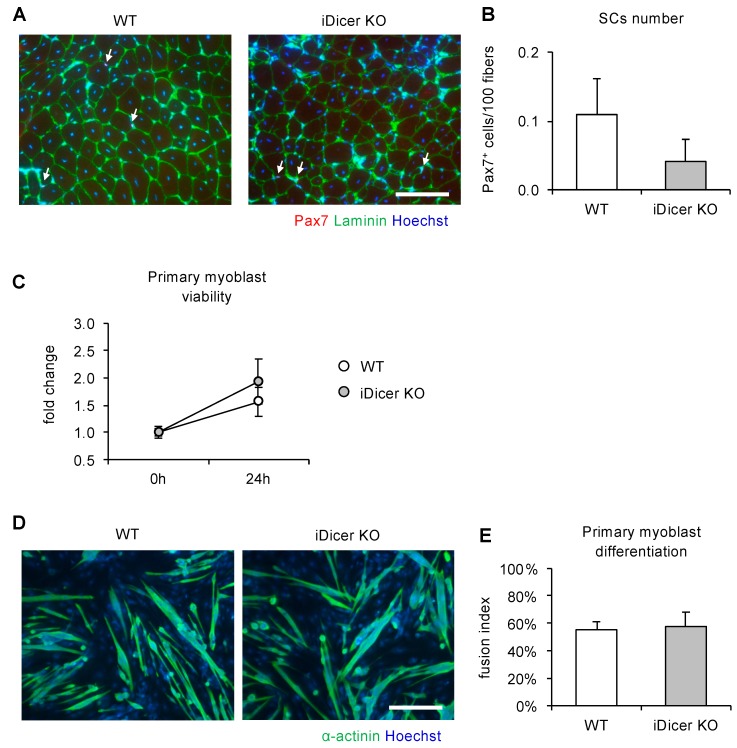
Satellite cell (SC) numbers and myogenic differentiation potential in primary myoblasts isolated from WT and iDicer KO mice (**A**,**B**). Immunofluorescence analysis revealed that the number of PAX7^+^ SCs in the iDicer KO mice was similar to that in the WT mice (*n* = 6–8) (**C**–**E**). There was no difference in the cell viability of the primary myoblasts (**C**) or their fusion indices (**D**,**E**) in the WT and iDicer KO mice (*n* = 3–4). Data are means ± SE. Scale bar = 100 µm.

Because the regenerative capacity of adult skeletal muscle largely depends on the functions of the resident muscle stem cells, such as muscle SCs, we investigated their numbers and the myogenic differentiation potential of primary myoblasts isolated from iDicer KO mice. Fourteen days after CTX injection, the number of PAX7^+^ cells on the cryosections did not differ in the WT and iDicer KO mice (Figure 3A,B). Furthermore, the cell viability and fusion index of the primary myoblasts isolated from the iDicer KO mice with tamoxifen injection were similar to those of the WT mice (Figure 3C–E).

## 3. Discussion

The tamoxifen-inducible knockout of Dicer in adult mice impaired the skeletal muscle regeneration that occurred in response to CTX injury (Figure 2). However, we found no reductions in the PAX7^+^ cell numbers, cell viability, or the myogenic differentiation potential of the primary myoblasts isolated from the iDicer KO mice (Figure 3). These data suggest that Dicer plays a prominent role in skeletal muscle regeneration in vivo. However, the molecular mechanism by which Dicer regulates skeletal muscle regeneration in this model is still unclear.

Recent studies have demonstrated that multiple miRNAs act as key regulators of skeletal muscle regeneration in adult mice [20,21,22,23,24]. For example, miR-26a, which is specifically expressed in skeletal muscle, is upregulated during muscle differentiation in vitro and muscle regeneration in vivo [10,21]. The inhibition of miR-26a with a miRNA decoy in vivo delayed muscle regeneration, indicating that miR-26a promotes muscle differentiation and regeneration [21]. It has also been shown that SC-specific *Dicer* knockout causes the apoptosis of SCs because they spontaneously leave the quiescent state, which results in severely impaired skeletal muscle regeneration after injury [25]. We also demonstrated that the CSA of regenerating fibers was reduced in the iDicer KO mice (Figure 2). Taken together, our data demonstrate the importance of miRNAs in skeletal muscle regeneration in mice.

Because the proliferation and differentiation of myoblasts are essential for skeletal muscle regeneration, we isolated the primary myoblasts from the skeletal muscles of iDicer KO mice, and determined the proliferation and myogenic differentiation potential of these cells. Surprisingly, there were no clear differences in cell viability or differentiation capacity of the primary myoblasts isolated from WT and iDicer KO mice (Figure 3C,D). Although the proliferation and differentiation of SCs are predominant factors in muscle repair, other cell types also affect the regeneration process [26]. Joe et al., [27] and Uezumi et al., [28] identified mesenchymal progenitors, called “fibro-adipogenic progenitors”, that enhance the myogenic differentiation of muscle stem cells and muscle regeneration [27,29]. Another recent work demonstrated that an interaction between capillary endothelial cells and SCs controls SC functions through a Notch-signaling-mediated direct cell–cell interaction [30]. Future research with mice in which individual miRNAs are cell-type-specifically knocked out may provide greater insight into the functions and regulatory mechanisms of miRNAs in muscle regeneration.

We found that muscle regeneration was disrupted in the iDicer KO mice, whereas expressions of muscle-enriched miRNAs (miR-1, miR-133a, miR-206 and miR-26a) and other miRNAs (miR-15b, miR-20a, miR-119a-3p, miR-214, miR-146a, miR-21 and miR-24) that were reported to be upregulated during muscle regeneration [31], were reduced only ~20−40% in the TA muscle of the iDicer KO mice compared with those in the WT mice (Figure 1B). It is not clear how the slight changes in miRNAs could contribute to the delayed regeneration in the iDicer KO mice. It may be possible that the global reduction in miRNAs is enough to disrupt a regulatory network of miRNAs and to delay the myofiber regeneration in vivo.

Our data showed that the muscle-enriched miRNAs were highly stable in the TA muscle of the iDicer KO mice (Figure 1B), which is consistent with our previous report [19]. Similarly, recent data on tamoxifen-inducible, skeletal muscle-specific Dicer knockout (HSA-MCM; Dicer^fl/fl^) mice showed that the expression levels of miRNAs including miR-1, miR-133a and miR-206 were slightly affected by the Dicer depletion [32]. Several lines of evidence indicate that the mature miRNAs expressed in liver, heart, and neuron are also stable in vivo [33,34,35]. A pulse-chase approach and high-throughput sequencing of miRNAs revealed the production and turnover rates of miRNAs in vitro [36,37], whereas miRNA turnover in vivo and its regulatory mechanism are less well understood. It will be of interest in future works to investigate how miRNA turnover is regulated in vivo.

## 4. Materials and Methods

### 4.1. Animal Experiments

All mice were maintained in temperature-controlled quarters (21 °C) under a 12 h light–dark cycle and provided with a standard chow diet. The generation of tamoxifen-inducible Dicer knockout (CAG-Cre^ERT2^, Dicer^fl/fl^) mice has been described previously [19]. The mice were maintained in a B6 background and intraperitoneally injected with 1 mg of tamoxifen at 8 weeks old for 5 consecutive days. The tibialis anterior (TA) muscles of 9-week-old wild-type (WT) and iDicer KO mice were injected with cardiotoxin (CTX) from *Naja pallida* (Latoxan, Portes-lès-Valence, France) with a 27-gauge needle. The TA muscles were harvested 7 and 14 days after CTX injury, frozen in liquid nitrogen, and stored at −80 °C. The animal protocols were approved by the Animal Care and Use Committees of Waseda University, Japan (numbers: 2017-A103a, 2018-A122, 2019-108).

### 4.2. qPCR

Total RNA was extracted with Isogen II (Wako Chemicals, Osaka, Japan), and 1 µg of total RNA was converted to cDNA with ReverTra Ace reverse transcriptase (Toyobo, Osaka, Japan). Real-time PCR was performed with Thunderbird^®^ SYBR^®^ qPCR Mix (Toyobo), with gene-specific primers. The following primers were used for real-time PCR: Dicer, 5′-CACACGCCTCCTACCACTACAACAC-3′ and 5′-CCGTGGGTCTTCATAAAGGT-3′; glyceraldehyde 3-phosphate dehydrogenase (GAPDH), 5′-AAATGGTGAAGGTCGGTGTG-3′ and 5′-TGAAGGGGTCGTTGATGG-3′. For the real-time PCR analysis of mature miRNA expression, the TaqMan^®^ MicroRNA Reverse Transcription Kit and TaqMan^®^ MicroRNA Assays (Applied Biosystems, Foster City, CA, USA) were used, according to the manufacturer’s protocols [38,39].

### 4.3. Histological Analysis

Cross-sections of frozen TA muscle were stained with H&E and immunofluorescence. The cross-sections were incubated with the following antibodies: anti-PAX7 (DSHB; University of Iowa, Iowa City, IA), anti-laminin (10765; Cappel Reseach Reagent, Turnholt, Belgium), and Hoechst 33342 (Invitrogen, Carlsbad, CA). The secondary antibodies were Alexa-Fluor-594-conjugated anti-mouse IgG antibody for PAX7 and Alexa-Fluor-488-conjugated anti-rabbit IgG antibody for laminin (Jackson ImmunoResearch Laboratories, West Grove, PA, USA). The cross-sectional area (CSA) was quantified using ImageJ software (National Institutes of Health, Bethesda, MD, USA).

### 4.4. Isolation and Culture of Primary Myoblasts

Primary myoblasts were isolated as previously described [40,41]. Briefly, after tamoxifen injection, the gastrocnemius, TA, and quadriceps muscles were collected and digested with 0.2% collagenase type II (Worthington Biochemical Corporation, Freehold, NJ, USA) at 37 °C for 30 min. After incubation, the muscle slurries were triturated with an 18-gauge needle and then incubated again at 37 °C for 15 min. The muscle mixtures were filtered sequentially through 100 μm and 40 μm filters (Falcon, Sunnyvale, CA, USA), and then centrifuged at 200× *g* for 5 min. The cells were resuspended in growth medium containing Ham’s F-10 Nutrient Mix (Thermo Fisher Scientific, Waltham, MA, USA) supplemented with 20% fetal bovine serum, 1% penicillin–streptomycin, and 2.5 ng/mL basic fibroblast growth factor (G5071; Promega, Madison, WI, USA), and seeded in gelatin-coated dishes. To evaluate cell viability, 3000 cells/well were seeded in 96-well plates and a CellTiter-Glo^®^ 2.0 Cell Viability Assay (Promega) was performed, according to the manufacturer’s protocol. To induce cell differentiation, the myoblasts in the 96-well plate at 80–90% confluence were switched to differentiation medium (DM; DMEM supplemented with 2% horse serum). The DM was changed daily for 5 days. After differentiation, the cells were immunofluorescently stained with Hoechst 33342 and anti-α-actinin antibody (A7732; Sigma-Aldrich, St Louis, MO, USA) to identify nuclei and differentiated myotubes, respectively. The secondary antibody for anti-α-actinin was an Alexa-Fluor-488-conjugated goat anti-mouse IgG_1_ antibody (Jackson ImmunoResearch Laboratories). The fusion index was calculated manually as the ratio of the number of nuclei within the α-actinin-positive myotubes with more than two nuclei to the total number of nuclei, using ImageJ software (LOCI, University of Wisconsin, Madison, WI, USA).

## Abbreviations

SC	Satellite cell
miRNA	microRNA
TA	Tibialis anterior
WT	Wild-type
CSA	Cross-sectional area
CTX	Cardiotoxin
